# Comparative Analysis of Glandular and Extraglandular Manifestations in Primary and Secondary Sjögren’s Syndrome: A Study in Two Academic Centers in North-East Romania

**DOI:** 10.3390/diagnostics14212367

**Published:** 2024-10-23

**Authors:** Alexandru Lodba, Codrina Ancuta, Diana Tatarciuc, Angela Ghiorghe, Luciana-Oana Lodba, Cristina Iordache

**Affiliations:** 1 Faculty of Dental Medicine, Grigore T. Popa University of Medicine and Pharmacy, 700115 Iasi, Romania; lodba.alexandru@d.umfiasi.ro (A.L.); diana.tatarciuc@umfiasi.ro (D.T.); cristina.ghiorghe@umfiasi.ro (A.G.); marina.iordache@umfiasi.ro (C.I.); 2 Department of Rheumatology, Faculty of Medicine, Grigore T. Popa University of Medicine and Pharmacy, 700115 Iasi, Romania; 3 Department of Medicine Specialties, Faculty of Medicine, Grigore T. Popa University of Medicine and Pharmacy, 700115 Iasi, Romania; 4 Cariology and Restorative Odontotherapy Discipline, Faculty of Dental Medicine, Grigore T. Popa University of Medicine and Pharmacy, 700115 Iasi, Romania; 5 LC Dental Orthoffice S.R.L., 725300 Gura Humorului, Romania; lucianacondur@gmail.com; 6 Ergonomics Discipline, Faculty of Dental Medicine, Grigore T. Popa University of Medicine and Pharmacy, 700115 Iasi, Romania

**Keywords:** primary Sjogren’s syndrome, secondary Sjogren’s syndrome, salivary disfunction, oral health

## Abstract

Background: This study investigates the clinical characteristics and differences between primary Sjögren’s Syndrome (pSS) and secondary Sjögren’s Syndrome (sSS) in a cohort of 50 patients. Methods: Conducted across two academic facilities in North-East Romania, the study emphasizes the importance of glandular and extraglandular manifestations, focusing on salivary flow rates, pH levels, and buffer capacity. Patients were diagnosed using the 2016 ACR-EULAR classification criteria, with a detailed examination including salivary tests, biopsies, and antibody presence. Results: The findings highlight significant differences between pSS and sSS, particularly in salivary function, with pSS patients exhibiting more severe glandular dysfunction. The study also notes a higher prevalence of inflammatory joint involvement in sSS patients, often associated with rheumatoid arthritis. Statistical analysis revealed correlations between salivary parameters and disease progression, underscoring the necessity of tailored treatment strategies. The research suggests that lower salivary flow rates and altered pH levels in pSS patients contribute to compromised oral health, including increased dental cavities and periodontal disease. Conclusions: The study’s results contribute to a deeper understanding of Sjögren’s Syndrome and reinforce the need for multidisciplinary management to address both systemic and oral health complications in these patients.

## 1. Introduction

Sjögren’s Syndrome (SS) is a systemic autoimmune disorder characterized by lymphocytic infiltration of exocrine glands, leading to progressive glandular dysfunction and various systemic manifestations [1]. The disease is complex and multifaceted, requiring a comprehensive, multidisciplinary approach to diagnosis, treatment, and management [2]. The syndrome often presents with symptoms that overlap between the two domains, such as joint pain, stiffness, and more severe manifestations like systemic vasculitis and interstitial lung disease [3].

The pathophysiological mechanisms of SS—such as chronic inflammation, autoantibody production, and glandular dysfunction—are closely related to treatment strategies that often involve medications affecting both rheumatological and dental health [4,5].

The treatment of SS is primarily symptomatic and focuses on managing the glandular and systemic manifestations of the disease. In more severe cases, immunosuppressive therapies may reduce the inflammatory response and preserve glandular function [6]. Rheumatological manifestations of SS are typically managed with nonsteroidal anti-inflammatory drugs (NSAIDs), corticosteroids, and disease-modifying antirheumatic drugs (DMARDs) [7,8]. In patients with more severe systemic involvement, such as vasculitis or interstitial lung disease, immunosuppressive therapies such as rituximab, a B-cell depleting agent, may be required [9,10,11,12].

Sjögren’s Syndrome treatment guidelines are developed by major organizations like the American College of Rheumatology (ACR), the European League Against Rheumatism (EULAR), and the British Society for Rheumatology (BSR). These guidelines are based on the latest research, expert consensus, and clinical experience. ACR and EULAR use classification criteria, such as objective tests, salivary gland biopsy, and clinical signs, to ensure accurate diagnosis and stratify patients for appropriate treatment. BSR guidelines also emphasize clinical assessment, serologic testing, and biopsy when necessary. These guidelines are a global standard for managing Sjögren’s Syndrome [13,14,15].

SS prevalence varies widely depending on the population studied and the diagnostic criteria used. Estimates suggest that SS affects between 0.1% and 4% of the population, with a higher prevalence in women than in men [16,17,18,19]. The exact etiology of SS remains unclear, but it is believed to involve a combination of genetic, environmental, and hormonal factors [19,20,21].

The term “secondary SS” (sSS) has been used to describe patients with SS in combination with another systemic autoimmune disease. At the same time, the term “primary SS” (pSS) was introduced for that category of patients for whom SS is the only diagnosis [4,7]. However, this terminology is under debate, and standardized protocols clarifying when or why these terms should be used are lacking. Secondary SS is mainly used in patients with concomitant systemic autoimmune diseases, such as rheumatoid arthritis [22], multiple sclerosis [23], or systemic lupus erythematosus (LES) [24], and is rarely used in patients with concomitant autoimmune organ diseases (e.g., autoimmune thyroiditis, primary biliary cholangitis, or autoimmune hepatitis) [25].

Oral health implications are significant, with chronic xerostomia—stemming from salivary gland dysfunction—leading to an increased risk of dental cavities, particularly in areas typically resistant to decay, such as cervical and smooth surfaces. This condition is also associated with periodontal disease, fungal infections, and difficulties in speaking, swallowing, and altered taste perception, as well as burning mouth syndrome and challenges in wearing dentures [12].

The management of these dental and oral issues is complicated by the underlying autoimmune nature of the disease, which requires careful coordination between dental and medical care providers [26,27]. The intersection of rheumatology and dentistry in the context of SS is not merely coincidental but an intrinsic feature of the syndrome [28].

Distinguishing between primary and secondary Sjögren’s Syndrome is crucial for understanding their clinical implications, particularly in terms of salivary function and systemic involvement.

While the glandular manifestations of SS, such as decreased salivary flow, are well-documented, the relationship between salivary function and disease progression in both pSS and sSS requires further investigation. Understanding these differences could enhance diagnostic accuracy and treatment approaches. The objectives of this study are to evaluate the salivary flow rate under both stimulated and unstimulated conditions, along with the salivary pH and buffer capacity, and their association with disease progression in SS. Additionally, we aim to conduct a comparative analysis between pSS and sSS based on antibody presence and treatment modalities.

Despite significant advancements in the understanding of Sjögren’s Syndrome (SS), there remains a lack of comprehensive data comparing the glandular and extraglandular manifestations between pSS and sSS. This distinction is critical for optimizing patient management, yet few studies have explored these differences in a clinically meaningful way. The purpose of this study is to address this gap by systematically analyzing both glandular and extraglandular manifestations in patients with pSS and sSS. Understanding these differences will enhance diagnostic accuracy and guide treatment strategies tailored to each subgroup.

Our study aims to explore the glandular and extraglandular manifestations in patients with primary and secondary Sjogren’s syndrome, with the goal of enhancing understanding and implementing effective patient management strategies.

## 2. Materials and Methods

We performed a retrospective, cross-sectional study in two academic facilities in North-East Romania (the adult Dental Clinic at St. Spiridon’s Hospital Iasi and the 2nd Rheumatology Department, Clinical Rehabilitation Hospital, Iasi, Romania). This retrospective cross-sectional study was conducted from January 2022 to January 2024 and included 50 consecutive patients diagnosed with either pSS or sSS, all of whom had at least one monitoring visit during this period. Patients were categorized based on their diagnosis confirmed through salivary gland biopsy or positive anti-SSA/SSB antibodies. Patients exhibiting symptoms of xerostomia and xerophthalmia for at least 3 months, not attributable to other conditions were considered as inclusion criteria, and as an exclusion criteria, we considered patients with other autoimmune diseases unrelated to SS. We further evaluated the glandular manifestations as Unstimulated Whole Salivary Flow Rate (UWSFR), Stimulated Whole Salivary Flow Rate (SWSFR), Salivary pH and Salivary buffer capacity. Also, as extraglandular manifestations we considered the presence of autoimmune-associated conditions (e.g., rheumatoid arthritis, systemic lupus erythematosus) and the specific immunologic markers and the evaluation of systemic symptoms, including joint pain and fatigue.

Upon signing the informed consent and the hospital agreement to process data, participants filled out a questionnaire that included inquiries about gender, age, level of education, as well as any underlying medical conditions for which they are now receiving pharmacological treatments. Following the completion of the questionnaire, the patients underwent a comprehensive medical examination, including blood tests, salivary test, salivary gland biopsy, and X-ray examinations when necessary. The data were documented in the medical record appended to the previously filled-out questionnaire.

All patients fulfilled the American College of Rheumatology/European League Against Rheumatism (ACR/EULAR) classification criteria [29]. Thus, due to the similar characteristics of the two autoimmune diseases (sSS and pSS) and the possibility of diagnostic challenges, imaging modalities were used to confirm the erosive nature of joint involvement in patients with sSS.

The patients were studied according to their demographic data, laboratory parameters, associated diseases, treatment modalities, and salivary parameters. Organic manifestations (such as lung, kidney, cutaneous involvement, and lymphadenopathy) were defined according to the EULAR Sjögren’s Syndrome Disease Activity Index (ESSDAI) domains [14,30,31]. Laboratory parameters were determined as part of the general routine investigation and follow-up of the involved patients at the Department of Laboratory Medicine. C-reactive protein, rheumatoid factor IgM, and total immunoglobulin G concentrations were measured using the immunoturbidimetric method (XL1000 clinical chemistry analyzer, Erba-Lacherma, Brno, Czech Republic). Antinuclear antibodies (anti-Ro/SS-A, and anti-La/SS-B, IgG, complement C3 and C4, rheumatoid factor) were tested using ORGENTEC Alegria Automated Laboratory Diagnostics indirect immunofluorescence assay measurement. Salivary stimulated and unstimulated flow, pH saliva, and salivary buffer capacity were determined using GC Saliva-Check Buffer.

For the statistical analysis, SPSS software (version 24.0) was used to analyze variance (ANOVA), and two-sample *t*-tests were used. For discrete parameters the chi-square test was performed when the expected data of *p* values < 0.05 were considered statistically significant. Also, the resulting data were translated to graphical images using Origin 2021. All data and graphics were obtained by dividing the patient into two main groups: pSS patients and sSS patients.

## 3. Results

After studying the demographic and general data of the patients in the study, we observed that the majority were women, 88%, with a mean age of 54.6. The total number of patients taken into the study was 50, 27 were diagnosed with pSS, and 23 were diagnosed with sSS. The male/female ratio of the total patients taken into consideration was 6/44, where 20/44 (female) were diagnosed with sSS, and 24/44 (female) were diagnosed with pSS (Table 1). Patients diagnosed with sSS were also under observation for rheumatoid arthritis (RA), systemic lupus (SLE), Polymyositis (PM), Undifferentiated connective tissue disease (UCTD) (initially presented with clinical features not fully meeting criteria for a specific autoimmune disease at the time of diagnosis), and Psoriatic arthritis (PsA). In total, 23 (46%) patients were identified to have some extraglandular manifestations, such as autoimmune inflammatory disease or inflammatory joint involvement. They were further divided into different groups according to having sSS associated with rheumatoid arthritis (RA), Sjögren’s syndrome as an extraglandular manifestation (sSS, *n* = 15; 30%), or having LES associated with Sjögren’s syndrome (*n* = 4; 8%) or having other autoimmune associated diseases, such as Undifferentiated Connective Tissue Disease (*n* = 2, 4%), polymyositis (*n* = 1, 2%), and psoriatic arthritis (*n* = 1, 2%).

The other group consisted of patients having pSS (*n* = 27, 54%) and were analyzed for their glandular manifestations (unstimulated whole salivary flow rate (UWSFR) and stimulated whole salivary flow rate (SWSFR), salivary pH, buffer capacity, and also a salivary gland biopsy).

Our results indicate that patients with pSS exhibited significantly lower salivary flow rates and altered pH levels compared to those with sSS, which suggests a greater degree of glandular dysfunction in pSS (Table 2). Patients with pSS often reported more severe symptoms, including exacerbated xerophthalmia and fatigue compared to those with sSS. This disparity can impact patients’ quality of life and necessitates different management strategies.

### 3.1. Study Subjects and Glandular Manifestations of pSS and sSS

Identified and diagnosed patients with pSS or sSS were observed and tested for significant differences between salivary flow rate (UWSFR, SWSFR), salivary pH and buffer capacity. A comparison of the observed variables between pSS and sSS patients and the patient classification based on the symptom severity is shown in Table 1.

**Table 1:** Characteristics of pSS and sSS patients.

Parameter	pSS *n* = 27 (54%)	sSS *n* = 23 (46%)	pSS vs. sSS	*p* Value
Age	53.71 (27–72)	55.83 (37–71)	0.96	>0.05
Male	61.5 (59–66)	65 (64–66)	0.94	>0.05
Female	52.4 (27–72)	54.9 (37–71)	0.95	>0.05
UWSFR (mL/min)	0.22 (0–1.2)	0.24 (0.1–0.7)	0.91	<0.001
SWSFR (mL/min)	0.92 (0–2)	1.1 (0.1–2)	0.83	<0.001
pH	6.26 (5–7.2)	6.34 (5.5–7.2)	0.98	<0.001
buffer capacity	10.7 (6–12)	11 (4–12)	0.97	<0.001

Abbreviations: pSS, primary Sjögren’s Syndrome; sSS, secondary Sjögren’s Syndrome; UWSFR, unstimulated whole salivary flow rate; SWSFR, stimulated whole salivary flow rate. All presented data are expressed as median and interquartile range (IQR).

**Table 2:** Monitoring the main salivary parameters as a comparison for pSS and sSS patients.

Parameter	pSS *n* = 27 (54%)	Mean ± SD (mL/min)	sSS *n* = 23 (46%)	Mean ± SD (mL/min)
Low rate of UWSFR (lover than 0.4 mL/min)	21 (77.7%)	0.03 ± 0.092	17 (73.9)	0.02 ± 0.071
Very low rate of UWSFR (lower that 0.2 mL/min)	16 (59.25%)	0.07 ± 0.106	6 (26.08%)	0.05 ± 0.012
Low rate of SWSFR (0.9–1.1 mL/min)	11 (40.7%)	0.12 ± 0.096	6 (26.08%)	0.09 ± 0.26
Very low rate of SWSFR (0.5–0.9 mL/min)	8 (29.6%)	0.035 ± 0.26	2 (8.6%)	0.25 ± 0.26
pH (acid 5–6)	21 (77.7%)		3 (13%)	
pH (neutral 6.8–7.8)	6 (22.2%)		14 (60.8%)	
buffer capacity				
pH = 0–5	0		0	
pH = 6–9	5 (18.5%)		13 (56.5%)	
pH = 10–12	25 (92.6%)		16 (69.5%)	

**Figure 1:**
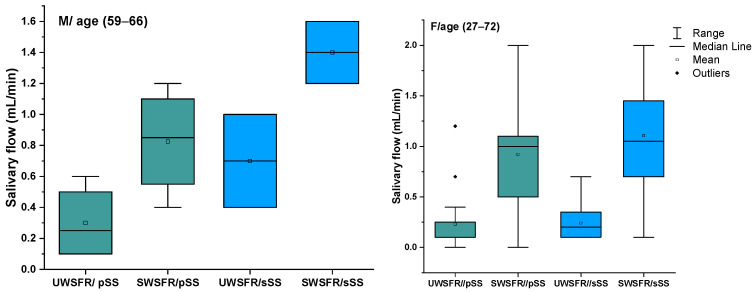
Comparison of UWSFR and SWSFR in pSS and sSS male and female. Abbreviations: pSS, primary Sjögren’s Syndrome; sSS, secondary Sjögren’s Syndrome, UWSFR, unstimulated whole salivary flow rate; SWSFR, stimulated whole salivary flow rate.

**Figure 2:**
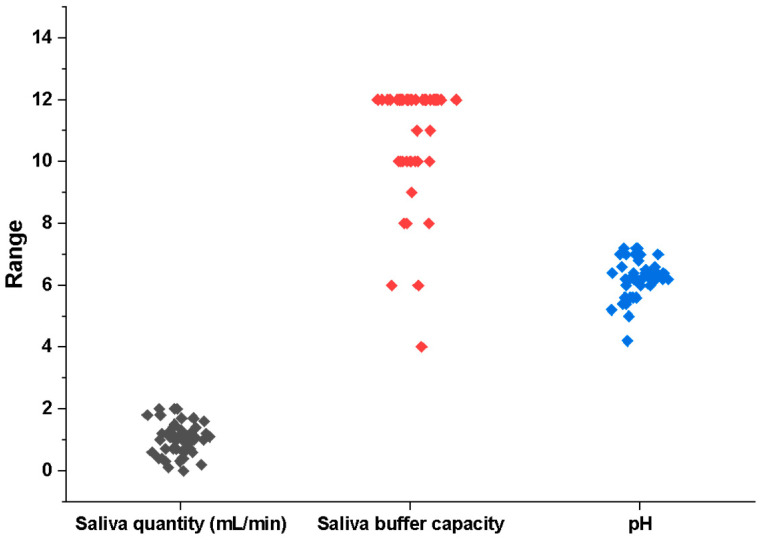
Correlation between salivary flow, buffer capacity and pH.

We reported statistically significant differences between the average values of the pH level, buffer capacity, and stimulated salivary flow, as can be seen in Figure 1. The quantity of stimulated saliva at 5 min is not significantly different between pSS and sSS, showing the same glandular reactivity in these two pathologies, respectively, 0.22–0.24 units. The buffer capacity of the stimulated saliva revealed a high number of patients, with the value 12 (38 out of 50) showing a significantly elevated buffer capacity, which may be related to the acidic pH values associated with a higher incidence of dental cavities in these patients (Figure 2).

### 3.2. Salivary Gland Biopsy as a Diagnosis Confirmation

A total of 27 out of 50 patients included in our research were diagnosed with pSS, and 21 patients underwent biopsy in the office setting under local anesthesia. No adverse events or complications were encountered during the procedure. No patients reported permanent complications after minor salivary gland biopsy. All the biopsies yielded adequate tissue samples for pathologic analysis; none of them were considered as inadequate. Of the 21 successful biopsies, 20 (95.2%) were suggestive of SS based on focus score. Of these, 14 patients (66%) subsequently started immunomodulatory therapy by their rheumatologist due to the multifactorial symptomatology. Salivary gland pain was the only symptom associated with a positive biopsy result, and seropositivity for any autoantibody and positive ANA were associated with a positive biopsy result [32].

### 3.3. Study Subjects and Extraglandular Manifestations of sSS

**Figure 3:**
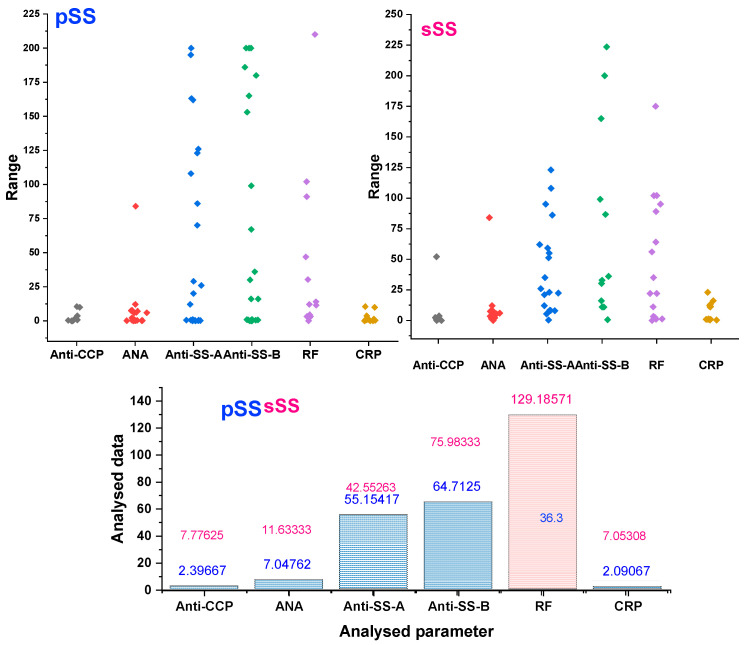
Comparative column scatter graphic of sSS and pSS values of Anti-CCP, ANA, Anti-Ro/SS-A, Anti-La/SS-B, RF and CRP and overlapped statistycal mean data.

The patients exhibited several extraglandular symptoms associated with sSS. Nevertheless, the sSS was strongly associated with RA (*n* = 15, 30%), SLE (*n* = 4, 8%), undifferentiated connective tissue disease (*n* = 2, 4%), polymyositis (*n* = 1, 2%) and psoriatic arthritis (*n* = 1, 2%). Table 3 shows the main characteristics of patients with sSS and the laboratory parameters evaluated (Figure 3).

**Table 3:** Characteristics of patient with pSS and sSS.

Parameter	pSS *n* = 27	Mean ± SD	sSS *n* = 23	Mean ± SD
Anti-CCP positivity > 20 U/mL	12 (44.4%)	2.39 ± 0.37	16 (69.5%)	3.19 ± 0.32
RF positivity > 20 UI/mL	7 (25.9%)	7.05 ± 1.7	21 (91.3%)	5.21 ± 0.23
ANA positivity > 1.2	16 (59.2%)	5.5 ± 0.7	16 (69.5%)	3.7 ± 0.21
Anti-Ro/SS-A positivity > 22 U/mL	15 (55.55%)	6.7 ± 0.81	12 (52.17%)	5.25 ± 0.23
Anti-La/SS-B positivity > 25 U/mL	15 (55.55%)	4.08 ± 0.61	12 (52.17)	6.3 ± 0.11
CRP positivity > 1 mg/dL	5 (18.5%)	0.2 ± 0.039	14 (60.86%)	0.3 ± 0.02
Polyarthritis	3 (11.1%)		21 (91.3%)	
Associated autoimmune diseases	4 (14.8%)		3 (13.04%)	
Associated non-autoimmunedisease	3 (11.1%)		4 (17.39%)	

Where: CCP: cyclic citrullinated peptide, RF: rheumatoid factor, ANA: antinuclear antibody, CRP: C-reactive protein, IgG. The laboratory test results are the median value determined in each subgroup. Reference ranges: anti-CCP < 25 U/mL, RF: <14 IU/mL, anti-Ro/SS-A: <10 U/mL, anti-La/SS-B < 10 U/mL, CRP: <5 mg/L.

### 3.4. Treatment

Table 4 summarizes the medications utilized for the treatment and control of the condition of patients involved in this study. In the group of individuals with sSS, the administration of methotrexate and antimalarial medications was shown to be much more common compared to other therapies. The individuals with pSS overlapped with sSS required glucocorticoids more often during the progression of their condition. The utilization of Methotrexate in treatment was progressively implemented, particularly for individuals exhibiting multiple inflammatory joint symptoms or overlapped autoimmune conditions. However, Azathioprine, a kind of immunosuppressive medicine, was only used in individuals diagnosed with no concrete response to other medications for rheumatoid arthritis. Furthermore, a few patients were receiving monoclonal antibody (Belimumab) or anti-tumor-necrosis factor (TNF)-alpha therapies [33]. However, there is still a lack of evidence about their progression.

**Table 4:** Medication of the patients with sSS taken into study.

Medication	sSS *n* = 23
Glucocorticoid	10%
Methotrexate	38%
Antimalarials	22%
Azathioprine	6%
Oral-health-supportive medication	40%
Belimumab	1%
Rituximab	3%
anti-tumor-necrosis factor (TNF)-alpha agents [etanercept (E), infliximab (I), adalimumab (A), certolizumab pegol (C), and golimumab (G)]	4%

The treatment employed for pSS patients was oral-health supportive medication, high oral hygiene, and oral rehabilitation.

## 4. Discussion

Our study aimed to study glandular and extraglandular manifestations in patients diagnosed with pSS or sSS and to draw a border between symptoms. We evaluated 50 patients, of which 44 were women and only 6 were men, with 88% of females with a mean age of 54.6, which is in concordance with literature data where women cases are more frequent. Most studies reported in the literature only analyzed either sSS [13,34,35] or pSS [36,37], but very few analyzed mirroring the symptoms and studying the same parameters. We observed a gender difference with strong female predominance; this gender disparity is attributed to several factors, including hormonal influences and genetic predispositions that increase susceptibility to autoimmune conditions in women [38].

We assessed both unstimulated whole salivary flow rate (UWSFR) and stimulated whole salivary flow rate (SWSFR) in our study, finding that our results are consistent with existing literature, which indicates that UWSFR typically ranges from 0.4 to 0.9 mL/min, and SWSFR from 0.4 to 0.6 mL/min. Notably, a significant proportion of our patients with primary Sjögren’s Syndrome exhibited low UWSFR, underscoring the severity of glandular dysfunction associated with this condition. Our results show a statistically significant difference in both unstimulated (UWSFR) and stimulated (SWSFR) salivary flow rates between patients with pSS and sSS (*p* < 0.001). However, while this difference is statistically significant, its clinical implications may vary. The difference in flow rates, although measurable, might not always translate into significant clinical symptoms or outcomes for all patients. Further research is needed to determine how these variations in salivary flow affect long-term clinical management. These percentages are higher than those observed in patients with sSS, validating the intense glandular degradation in patients with pSS. Also, these results are in accordance with the Sjögren Big Data Project, a study including a large group of Sjögren’s syndrome patients from different countries, where Brito-Zerón et al. found that 75.2% of the patients had a reduced saliva production while at rest [39].

A significant proportion of patients with secondary Sjögren’s Syndrome demonstrated a buffering capacity indicating their efforts to manage the acidic environment in the oral cavity. This highlights the challenges faced by patients with primary Sjögren’s Syndrome in maintaining oral health. Also, these data suggest that the ability of saliva to protect dental structures from acid attacks is diminished. Approximately 90% of the participants had a very low and acidic pH levels in their recorded salivary samples. The findings indicating low values for salivary pH, buffer capacity, and secretory flow salivation provide evidence of compromised oral health. Therefore, the data collected shows a higher occurrence of dental cavities, additionally, an equal number of individuals also experienced occurrences of periodontal disease or edentation.

We further analyzed the data for patients with secondary Sjögren’s Syndrome (sSS), observing that a significant majority exhibited inflammatory joint involvement, including rheumatoid arthritis and other associated autoimmune diseases, as well as a proportion with non-autoimmune conditions. Our results suggest that high anti-CCP and high RF levels in sSS patients with inflammatory joint pain indicate progression toward rheumatoid arthritis, which is in correlation with data from other studies were the anti-CCP antibody titer was strongly associated with the progression to RA [40].

In terms of treatment, we observed that a significantly larger proportion of sSS diagnosed patients require methotrexate treatment (38%), antimalarials (22%), and glucocorticoids (10%). Most of the patients with RA or SLE are on double therapy (methotrexate + antimalarials or antimalarial + azatioprine) or, in some extreme cases (3%), on rituximab and 1% on belimumab or 4% on TNF-alpha agents.

This might be attributed to the fact that glandular symptoms in primary Sjögren’s syndrome (pSS) typically do not require systemic therapy, with 40% of cases being managed with oral-health supportive medicine. Additionally, the pain and inflammation experienced in non-erosive polyarthritis are generally less frequent compared to those seen in RA. Azathioprine and antimalarials are often employed in individuals with secondary Sjögren’s syndrome (sSS) [40], indicating that these immunomodulatory drugs are mostly utilized to treat moderate cases of RA associated with sSS. Short-term treatment with glucocorticoids and oral health care were employed for pSS patients with an improvement on the unstimulated salivary flow, which is also confirmed by the literature [10]. Not unexpectedly, the treatment with methotrexate was consistently and considerably higher in patients with sSS, and these patients showed a positive response to the treatment in terms of improved sympthomatology and an increase in life quality of the patients under treatment. The observed differences in glandular dysfunction between pSS and sSS highlight the need for tailored management strategies, as patients with pSS may require more aggressive intervention to address their salivary dysfunction. The response to treatment can vary significantly between pSS and sSS patients. For example, pSS patients often require more aggressive treatment for glandular dysfunction and related systemic symptoms, while sSS patients may benefit from managing the underlying autoimmune disease, which can simultaneously alleviate symptoms of Sjögren’s Syndrome.

This study has several strengths, including its comprehensive assessment of both glandular and extraglandular manifestations in a well-defined cohort of patients with primary and secondary Sjögren’s Syndrome. The use of standardized testing methods for salivary parameters and the inclusion of a diverse patient population strengthen the validity of our findings. However, there are some limitations to consider. The study’s retrospective design may introduce selection bias, and the sample size, while adequate for preliminary findings, may limit the generalizability of the results. Additionally, the lack of longitudinal data restricts our ability to draw conclusions about the progression of symptoms over time. Future research should focus on longitudinal studies to assess the long-term progression of salivary dysfunction in SS patients and its impact on oral health. Furthermore, exploring the molecular mechanisms underlying the differences in glandular and extraglandular manifestations between pSS and sSS could provide valuable insights into targeted therapies.

## 5. Conclusions

The study design and findings emphasize the importance of performing oral health screening in patients diagnosed with primary Sjögren’s syndrome (pSS) and secondary Sjögren’s syndrome (sSS), as well as the necessity of continuous monitoring for these individuals. Also, our study underscores the critical differences between primary and secondary Sjögren’s Syndrome, emphasizing the need for differentiated clinical approaches based on the unique characteristics of each condition.

Future study should focus on determining the optimal method for assessing xerostomia, the dryness of the mouth, in patients with pSS. Additionally, conducting ultrasound scans of the main salivary glands should be given priority. This study is constrained by several limitations. Initially, the data were obtained retrospectively, indicating that they were collected from previous records and may not possess the same level of reliability as prospective data. Furthermore, disease activity ratings were not calculated because of the overlapping features of Sjögren’s syndrome (SS) and rheumatoid arthritis (RA). This could potentially impact the precision of the results.

## Data Availability

The original contributions presented in the study are included in the article, further inquiries can be directed to the corresponding author.
